# Cathepsin S Is More Abundant in Serum of *Mycobacterium avium* subsp. *paratuberculosis*-Infected Dairy Cows

**DOI:** 10.3390/metabo14040215

**Published:** 2024-04-11

**Authors:** Heidi C. Duda, Christine von Toerne, Lucia Korbonits, Andrea Didier, Armin M. Scholz, Erwin Märtlbauer, Stefanie M. Hauck, Cornelia A. Deeg

**Affiliations:** 1 Chair of Physiology, Department of Veterinary Sciences, Ludwig Maximilian University of Munich, D-82152 Martinsried, Germany; 2 Metabolomics and Proteomics Core, Helmholtz Center Munich, German Research Center for Environmental Health, D-85763 Neuherberg, Germanystefanie.hauck@helmholtz-munich.de (S.M.H.); 3 Chair of Hygiene and Technology of Milk, Department of Veterinary Sciences, Ludwig Maximilian University of Munich, D-85764 Oberschleißheim, Germany; a.stockmaierdidier@lmu.de (A.D.);; 4 Livestock Center of the Faculty of Veterinary Medicine, Ludwig Maximilian University of Munich, D-85764 Oberschleißheim, Germany; armin.scholz@lvg.vetmed.uni-muenchen.de

**Keywords:** *Mycobacterium avium* subsp. *paratuberculosis*, serum proteomics, quantitative label-free LC-MS/MS, immune system, host–pathogen response, Cathepsin S, bovine

## Abstract

*Mycobacterium avium* subsp. *paratuberculosis* (MAP) is the causative agent of bovine paratuberculosis, a chronic granulomatous enteritis leading to economic losses and posing a risk to human health due to its zoonotic potential. The pathogen cannot reliably be detected by standard methods, and immunological procedures during the infection are not well understood. Therefore, the aim of our study was to explore host–pathogen interactions in MAP-infected dairy cows and to improve diagnostic tests. Serum proteomics analysis using quantitative label-free LC-MS/MS revealed 60 differentially abundant proteins in MAP-infected dairy cows compared to healthy controls from the same infected herd and 90 differentially abundant proteins in comparison to another control group from an uninfected herd. Pathway enrichment analysis provided new insights into the immune response to MAP and susceptibility to the infection. Furthermore, we found a higher abundance of Cathepsin S (CTSS) in the serum of MAP-infected dairy cows, which is involved in multiple enriched pathways associated with the immune system. Confirmed with Western blotting, we identified CTSS as a potential biomarker for bovine paratuberculosis. This study enabled a better understanding of procedures in the host–pathogen response to MAP and improved detection of paratuberculosis-diseased cattle.

## 1. Introduction

Bovine paratuberculosis, also known as Johne’s disease, is a bacterial infectious disease caused by *Mycobacterium avium* subsp. *paratuberculosis* (MAP), an intracellular pathogen [1,2]. Paratuberculosis manifests as a chronic granulomatous enteritis, leading to high economic losses due to reduced milk production, premature culling and decreased slaughter value [3,4,5]. Transmission primarily occurs fecal–orally, whereby calves are more susceptible to infection in the first few months of life [6,7]. During a long asymptomatic subclinical phase, infected cattle shed the pathogen continuously or intermittently, posing a high risk of spreading within the herd [8]. Additionally, MAP is highly resistant to environmental influences, with survival over several months in fecal excretions and soil [9].

The detection of MAP-infected cattle is problematic due to the unreliability of rapid testing methods [10]. Standard diagnostic tests for identifying infected cattle can be divided into indirect and direct pathogen detection methods [10]. The common serology-based test is the detection of MAP-specific antibodies in an enzyme-linked immunosorbent assay (ELISA) [10]. The reliable detection of MAP-infected cattle in ELISA is restricted due to its limited sensitivity and specificity, and the absent seroconversion in the subclinical phase leads to a high rate of undetected diseased cattle [10,11,12]. The direct pathogen detection occurs through polymerase chain reaction (PCR) or cultivation [10,11]. The PCR method is also characterized by limited sensitivity and specificity [10], whereas the cultivation as a gold standard method reveals a high specificity [11]. However, the method is time-consuming due to the slow growth of MAP, requiring cultivation for at least 8 weeks [13].

MAP has been found in various human foods, including raw and pasteurized milk [14,15], infant formula [16] and meat [17], raising concerns about its zoonotic potential. The involvement of MAP in human autoimmune diseases like Crohn’s disease, type 1 diabetes and Hashimoto’s thyroiditis is being discussed [18,19,20,21,22].

There are several different control and eradication strategies to reduce the risk of within-herd transmission, especially to cattle of susceptible age, and eliminate infected animals [23,24]. Vaccination against bovine paratuberculosis also reduces shedding of MAP and thereby decreases prevalence [25]. However, vaccination interferes with an intradermal diagnostic test of bovine tuberculosis; therefore, the vaccine is not available in most European countries [26,27]. The strategy of eliminating infected animals through testing and culling is also being pursued but only results in a limited reduction in seroprevalence [28]. To avoid MAP exposure to calves and achieve a lower prevalence of MAP infections in dairy herds, hygiene and management strategies, e.g., restricting calves’ access to adult cattle and their feces, exist [29]. Nevertheless, the success of an eradication program based solely on hygiene and management strategies is limited [30].

Susceptibility and resistance to bovine paratuberculosis are associated with genetic variation in interferon gamma (IFNγ) and interleukin (IL) receptor 10, among other factors [31,32]. In a previous study, we detected two different immune phenotypes in cattle in Germany [33]. These phenotypes, characterized by different immune capacities, have previously been studied in detail with co-incubation of MAP and peripheral blood mononuclear cells (PBMCs) from cows with negative MAP infection status and different immunophenotypes [34]. In the PBMCs of the control group, we showed a higher abundance of IL-12-mediated signaling pathways, while we observed a higher abundance of CCR4-NOT transcription complex, subunit 1 in immune deviant cows, which may promote infectious diseases by repressing MHC class II expression [34,35]. In another co-incubation study of MAP and the PBMCs of cows with different MAP infection statuses, we detected a defensive immune response of MAP-infected cows to MAP, realized by an increased abundance of the involved proteins toll-like receptor 2 and major histocompatibility complex (MHC) class II [36]. These findings underscore the complexity of the immune response to MAP and highlight the need for further investigation into factors that contribute to the persistence of MAP in cattle.

Serum proteomics is currently often used for the identification of potential disease biomarkers and the detection of immunological processes during specific diseases [37,38]. Among others, this method already revealed IL-8 and Pentaxin as potential biomarkers for bovine tuberculosis in the serum of cattle [39]. In our proteomics study, we analyzed the serum of naturally MAP-infected dairy cows and healthy controls from the same infected herd and additional controls from another uninfected herd using label-free LC-MS/MS. The primary aim of our study was to discover proteomic changes in the serum of dairy cows with different MAP infection statuses to better characterize the host–pathogen response of cattle, particularly to MAP. In addition to this, this study aimed to identify a serum biomarker for the reliable detection of MAP-infected cattle for the control of bovine paratuberculosis.

## 2. Materials and Methods

### 2.1. Animals and Detection of MAP Infection Status

In this study, samples from 16 dairy cows from a MAP-infected farm were used. Serum, milk and fecal samples were collected. Different standard methods for detecting MAP were applied to categorize these cows into two groups based on their MAP infection status. Healthy controls (*n* = 7) showed no antibodies against MAP in ELISA with serum and milk samples (cattletype MAP Ab, Qiagen, Hilden, Germany; Indirect, IDVet, Grabels, France), and after cultivation of fecal samples on commercial Herrold’s Egg Yolk Agars (HEYM agar, Becton Dickinson, Heidelberg, Germany) for 12 weeks, no bacterial growth could be observed. Cows with positive results (*n* = 9) were grouped as MAP-infected cows. For mass spectrometry analysis, the serum of seven MAP-infected cows and seven healthy controls were used. All animals were from the same farm and were kept under the same environmental conditions. For additional mass spectrometry analysis with a further control group, the serum of 21 dairy cows from an uninfected farm was examined. All cows from this farm showed negative results in ELISA with serum and milk samples. Additionally, no positive samples could ever be detected in the regularly tested fecal samples and sock swab samples from this farm. To verify differences in CTSS expression in a Western blot analysis, five dairy cows from another infected farm were examined. The MAP status of these cows was determined by cultivating fecal samples on HEYM agar for 12 weeks and conducting ELISA with serum samples. In detail, two cows were categorized as healthy controls, and three cows were categorized as MAP-infected. A comprehensive overview of the MAP infection status of the cows in each group and a schematic of the experimental design are provided in Supplementary Table S1. Sampling of whole blood by venipuncture and experimental protocols were approved by the local authority, the Government of Upper Bavaria, permit no. ROB-55.2-2532.Vet_03-17-106. No experimental animals were used in this study. All animals were kept for the purpose of milk production. Permission from the dairy farms to use the blood samples from their animals for study purposes was obtained.

### 2.2. Sample Digestion for Differential Proteome Analysis

The serum was proteolyzed with trypsin using the PreOmics iST Kit (PreOmics GmbH, Martinsried, Germany) according to the manufacturer’s specifications. The resulting peptides were dried using SpeedVac and stored at −80 °C until mass spectrometric analysis.

### 2.3. Mass Spectrometric Analysis and Label-Free Quantification

500 ng of peptides per sample was analyzed in data-independent (DIA) mode on a Q Exactive HF mass spectrometer (Thermo Fisher Scientific Inc., Waltham, MA, USA) coupled online to a RSLC (Ultimate 3000, Thermo Fisher Scientific Inc.) HPLC system. Samples were automatically injected and loaded onto a nano-trap column (300-µm inner diameter (ID) × 5 mm, packed with Acclaim PepMap100 C18, 5 µm, 100 Å; LC Packings, Sunnyvale, CA, USA) before separation using reversed-phase chromatography (Acquity UPLC M-Class HSS T3 Column 75 µm ID × 250 mm, 1.8 µm; Waters, Eschborn, Germany) at 40 °C. Peptides were eluted from the column at a flow rate of 250 nl/min using increasing concentrations of ACN in 0.1% formic acid from 3 to 40% over a 45 min gradient. The DIA method consisted of a survey scan from 300 to 1500 *m/z* at 120,000 resolution and an automatic gain control (AGC) target of 3e6 or 120-millisecond maximum injection time. Fragmentation was carried out with higher energy collisional dissociation with a target of 3e6 ions, determined with predictive AGC. With an AGC target of 3e6 and automatic injection time, precursor peptides were isolated using 37 variable windows ranging from 300 to 1650 *m/z* at 30,000 resolution. Spectra were recorded in profile type with a normalized collision energy of 28.

DIA files were processed with Spectronaut (Version 18, Biognosys, Schlieren, Switzerland) as direct DIA analysis against the Ensembl cow database (Release 111, June 2023, 23,842 sequences) using BSG factory settings for Pulsar search. For DIA analysis, default settings were applied. For quantification, precursor filtering was set on Qvalue, the proteotypicity filter was on, and there was run wise imputing, protein LFQ method Quan2 algorithm, quantity MS level MS2, quantity type area, cross run normalization and mean top3 quantity. A ratio-based unpaired *t*-test was performed on the peptide level. The results were considered significant as Benjamini–Hochberg-corrected *p*-values at *q* ≤ 0.05.

### 2.4. Data Analysis

Volcano plots were generated using GraphPad Prism Software (version 5.04, GraphPad Software, San Diego, CA, USA). A pathway enrichment analysis was conducted with open-source software ShinyGO (version 0.77, http://bioinformatics.sdstate.edu/go/, accessed on 13 December 2023). The *p*-value cutoff (FDR) was set to 0.05, and the best-matching species (cow) was used. The *p*-value for the enrichment analysis was calculated using hypergeometric distribution followed by FDR correction. A pathway analysis with open-source software Reactome (version 87, https://reactome.org/, accessed on 13 December 2023) was performed on human orthologues gene names. Over-representation of pathways was determined with hypergeometric distribution corrected for FDR using the Benjamini–Hochberg procedure.

### 2.5. Western Blots

To 0.5 µL serum 1× Laemmli-buffer (50 mM tris-HCl, 1% SDS (AppliChem, Darmstadt, Germany), 10% glycerol (SERVA, Heidelberg, Germany), 4 mM 2-mercaptoethanol (Sigma-Aldrich, Taufkirchen, Germany), bromophenol blue (Sigma-Aldrich)) was added. Protein expression was analyzed separately for each biological replicate of controls (*n* = 9) and MAP-infected cows (*n* = 12), as well as for each technical replicate (*n* = 2). Proteins were separated with SDS-PAGE on 15% gels and blotted semi-dry onto PVDF membranes (GE Healthcare, Freiburg, Germany). To prevent unspecific binding, membranes were blocked with 4% PVP-T (Sigma-Aldrich). After washing, blots were incubated with a rabbit anti-bovine Cathepsin S polyclonal antibody (rabbit polyclonal, MyBioSource, San Diego, CA, USA; 1:600) at 4 °C overnight. After three washing steps with PBS-T (NaCl 136.9 mM (Sigma-Aldrich), Na_2_HPO_4_ × 2H_2_O 8.1 mM (AppliChem), KH_2_PO_4_ 1.4 mM (Sigma-Aldrich), KCl 2.6 mM (Sigma-Aldrich), 0.05% Tween 20 (AppliChem); pH 7.4), HRP-conjugated anti-rabbit IgG (Fc) secondary antibody (Bio-Rad, Feldkirchen, Germany; 1:10,000) preadsorbed with 5% heat-denatured bovine serum was used for incubation for one hour at room temperature. Following six washing steps, signals were detected with enhanced chemiluminescence on an Amersham Imager 680 (GE Healthcare). Quantification of Western blot signals was performed using ImageQuant TL v8.2.0 software (GE Healthcare). The Gaussian distribution was determined using the Kolmogorov–Smirnov test. Due to the non-normal distribution, statistics were performed using the Mann–Whitney test. The result was considered significant at *p* ≤ 0.05. Data were processed, analyzed and visualized with GraphPad Prism software (version 5.04, GraphPad Software).

## 3. Results

### 3.1. Serum Proteomics Reveals Differentially Abundant Proteins in Serum of MAP-Infected Dairy Cows When Compared to Two Healthy Control Groups from Farms with Divergent MAP Infection Status

**Figure 1:**
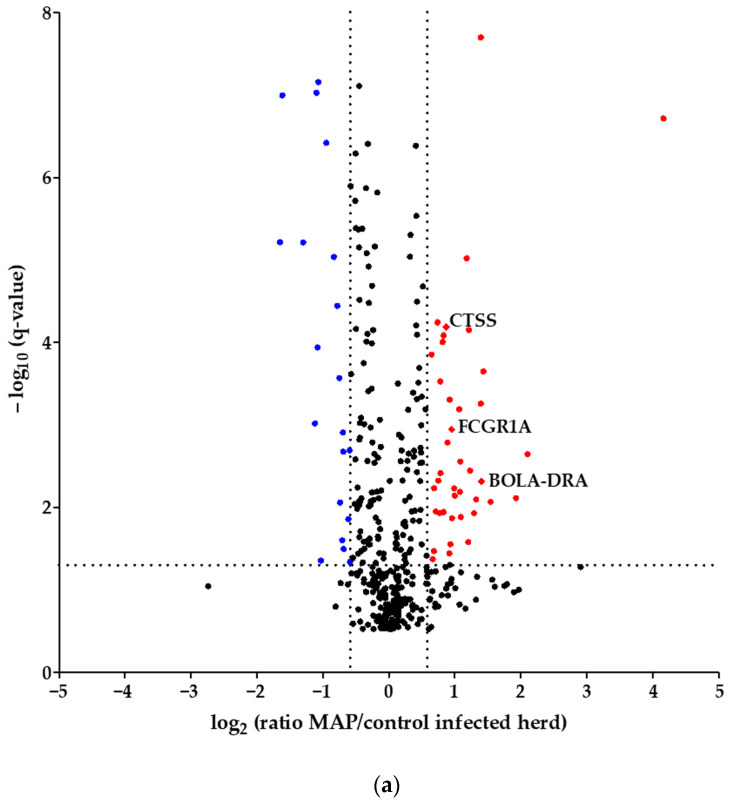

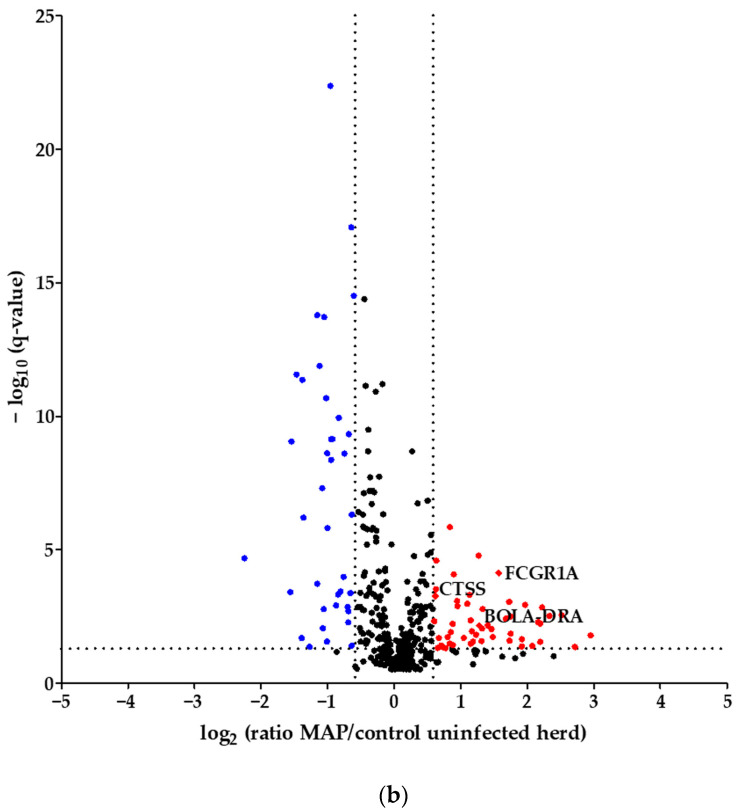
Volcano plot of all 394 identified proteins. (**a**) Comparing MAP-infected cows with healthy controls from the same dairy farm, 40 proteins showed significantly (*q* ≤ 0.05) higher abundance (fold change > 1.5, red dots), while 20 proteins showed significantly lower abundance (fold change < 0.$\bar{6}$, blue dots). (**b**) Fifty-two proteins were significantly (*q* ≤ 0.05) more abundant in MAP-infected cows compared to healthy controls from another dairy farm with MAP-uninfected status (fold change > 1.5, red dots), and 38 proteins were more abundant in the control group (fold change < 0.$\bar{6}$, blue dots). Significantly (*q* ≤ 0.05) more abundant proteins with strong associations to immune system pathways are displayed with their bovine gene names: Cathepsin S (CTSS), major histocompatibility complex, class II, DR alpha (BOLA-DRA) and Fc fragment of IgG, high-affinity Ia, receptor (FCGR1A).

Using quantitative mass spectrometric analysis, we identified a total of 394 proteins in bovine serum. Of these, 60 proteins were significantly (*q* ≤ 0.05) differently abundant between MAP-infected dairy cows (*n* = 7) and healthy controls (*n* = 7) from the same dairy farm. In detail, 20 proteins were more abundant in the control group, while 40 proteins showed increased abundance in MAP-infected cows (Figure 1a). Furthermore, an additional investigation was carried out with another control group comprising 21 cows from an uninfected herd. This revealed significant differential expression of 90 proteins (*q* ≤ 0.05). Of these, 38 proteins were upregulated in the control group, representing healthy cows from the uninfected herd, while 52 proteins showed upregulation in the MAP-infected cows (Figure 1b).

**Figure 2:**
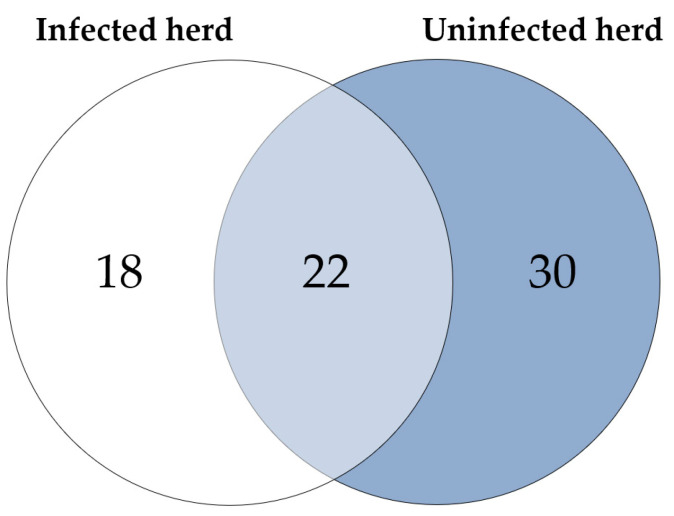
Significantly (*q* ≤ 0.05) more abundant proteins in MAP-infected cows: 40 proteins were more abundant compared to healthy controls from the same infected herd (indicated by white and light blue) and 52 compared to healthy controls from another uninfected herd (light blue and dark blue). Of these, 22 proteins overlapped when compared to both control groups (light blue).

Considering the proteins with higher abundance in MAP-infected dairy cows, 40 proteins were more abundant compared to healthy controls from the same infected farm, whereas 52 proteins showed higher abundance in comparison to healthy controls from another uninfected herd. Of these, 22 proteins overlapped when compared to both healthy control groups (Figure 2). The overlapping proteins included immunologically functional proteins such as “Cathepsin S” (CTSS), “major histocompatibility complex, class II, DR alpha” (BOLA-DRA) and “Fc fragment of IgG, high affinity Ia, receptor” (FCGR1A) (Figure 1a,b).

### 3.2. Proteins with Significantly Higher Abundance in MAP-Infected Cows Associate with Immune System Pathways

**Figure 3:**
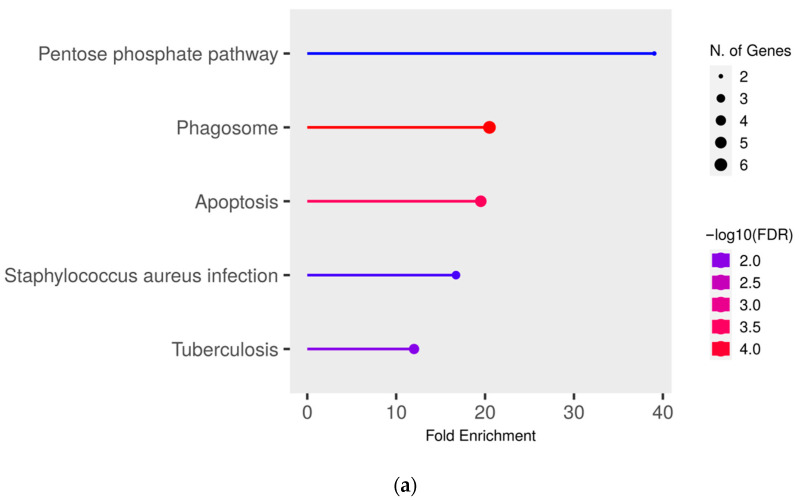

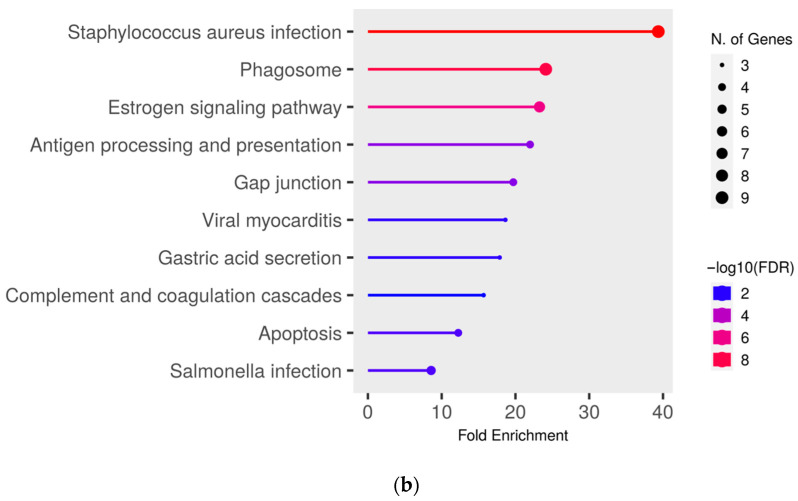
Pathway enrichment analysis of significantly (*q* ≤ 0.05) higher abundant proteins in MAP-infected cows compared to (**a**) healthy controls from the same infected herd and (**b**) healthy controls from another uninfected herd. Functional enrichment displays the ten most significant categories of all available gene sets. Hierarchical clustering was conducted using ShinyGO v0.77. The *y*-axis lists the assigned pathways in order of the enrichment of fold change ratio (FCR). The *x*-axis shows the FDR values for the enrichment of the respective pathways. The color chart shows fold enrichment for each pathway, with the size of dots corresponding to the number of genes associated with each pathway. Pathway enrichment analysis was conducted with bovine gene names.

To gain deeper insights into the functional effects of the differentially abundant proteins, we conducted a pathway enrichment analysis of all proteins with significantly (*q* ≤ 0.05) higher abundance (ratio MAP/control > 1.5) in MAP-infected cows compared to both healthy control groups using ShinyGO. This analysis revealed an enrichment of proteins in pathways associated with bacterial infections, such as “Tuberculosis” (compared to controls from the infected herd) (Figure 3a), “*Staphylococcus aureus* infection” (compared to both control groups) (Figure 3a,b) and “*Salmonella* infection” (compared to controls from the uninfected herd) (Figure 3b). Additional enrichment was observed in pathways belonging to the immune system such as “Phagosome” (compared to both control groups) (Figure 3a,b) and “Antigen processing and presentation” (compared to controls from the uninfected herd) (Figure 3b). There also was an enrichment of the pathways “Pentose phosphate pathway” (compared to controls from the infected herd) (Figure 3a) and “Apoptosis” (compared to both control groups) (Figure 3a,b).

**Figure 4:**
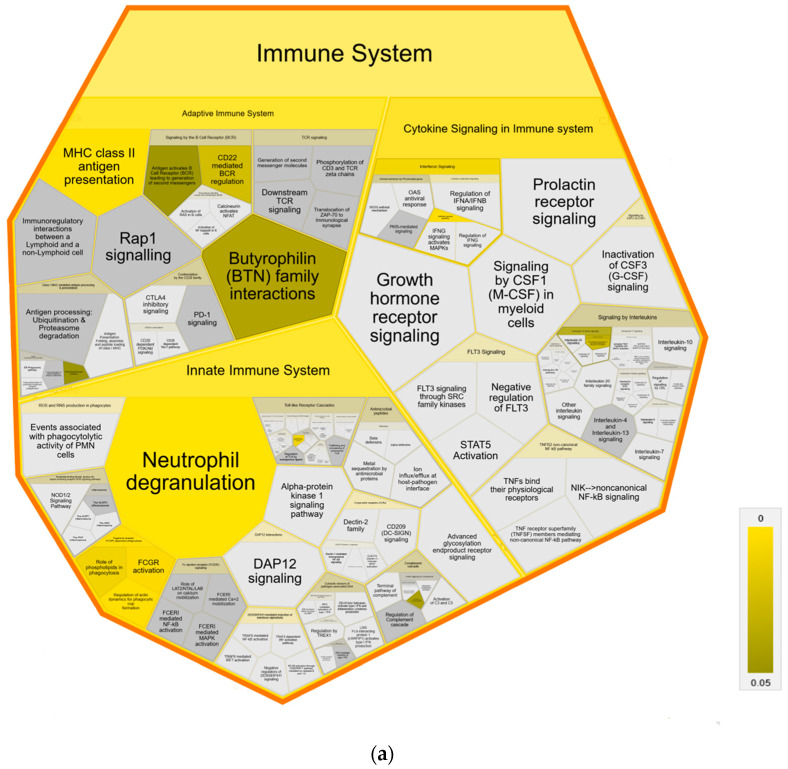

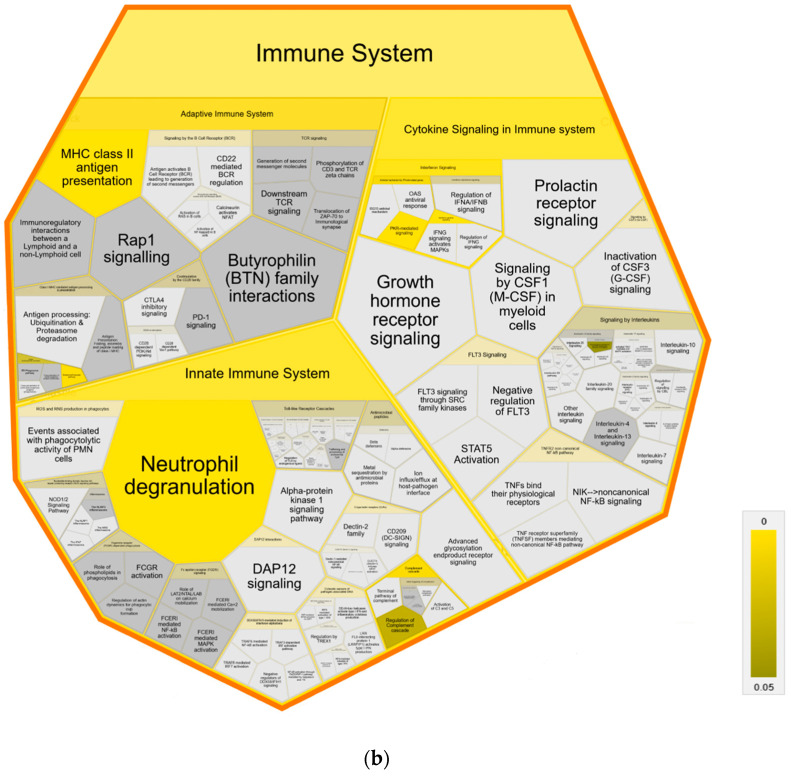
Voronoi diagram illustrating Reactome analysis results of enriched pathways in MAP-infected dairy cows compared to (**a**) healthy controls from the same infected herd and (**b**) healthy controls from another uninfected herd. The enlarged polygon of Reactome superpathway “Immune System” is shown. Pathway enrichment analysis was conducted with human orthologue gene names of proteins with significantly (*q* ≤ 0.05) higher abundance in MAP-infected dairy cows (ratio MAP/control > 1.5). Color intensity represents the *p*-value (*p* ≤ 0.05) of the statistical test for over-representation, as illustrated by the color bar. Polygons colored in dark grey visualize pathways without significant over-representation (*p* > 0.05), while light grey areas represent pathways without related proteins.

Further pathway analysis using Reactome confirmed an association with immune system pathways, showing enrichment in all three major subdomains: “Adaptive Immune System”, “Innate Immune System” and “Cytokine Signaling Immune System” of Reactome superpathway “Immune System” compared to both control groups (Figure 4). Specifically, we identified over-representation in pathways “MHC class II antigen presentation”, “Endosomal/Vacuolar pathway”, “Neutrophil degranulation”, “Gene and protein expression by JAK-STAT signaling after Interleukin-12” and “Interferon Signaling” with “Interferon gamma signaling” (Figure 4a,b). Pathways in MAP-infected cows that were enriched only in comparison to healthy controls from the same infected herd were “Butyrophilin (BTN) family interactions”, “Antigen activates B Cell Receptor (BCR) leading to generation of second messengers”, “CD22 mediated BCR regulation”, “Fcgamma receptor (FCGR) dependent phagocytosis” with “Role of phospholipids in phagocytosis”, “FCGR activation” and “Regulation of actin dynamic for phagocytic cup formation”, “Transfer of LPS from LBP carrier to CD14” and “Classical antibody-mediated complement activation” (Figure 4a). In contrast, compared to healthy controls from another uninfected herd, the pathways “PKR-mediated signaling” and “Regulation of Complement cascade” were enriched in MAP-infected cows. (Figure 4b).

### 3.3. CTSS Involved in Pathways with Strong Association to the Immune System

To delve deeper into the detected pathways, we examined the significantly upregulated proteins (*q* ≤ 0.05, ratio MAP/control > 1.5) in MAP-infected cows and the enriched pathways in which they were involved. We found that CTSS was participating in multiple pathways belonging to both the adaptive immune system, such as “MHC class II antigen presentation”, and the innate immune system, such as “Neutrophil degranulation” (Table 1). Additionally, CTSS was involved in the disease pathway “Tuberculosis” and programmed cell death pathway “Apoptosis”. The function of CTSS in multiple enriched pathways was observed in comparison to both control groups. All enriched proteins associated with the respective pathway and the total number of genes in each pathway are listed in Table 1.

### 3.4. Detection of Increased Abundance of CTSS in Serum of MAP-Infected Dairy Cows Using LC-MS/MS and Western Blotting

**Figure 5:**
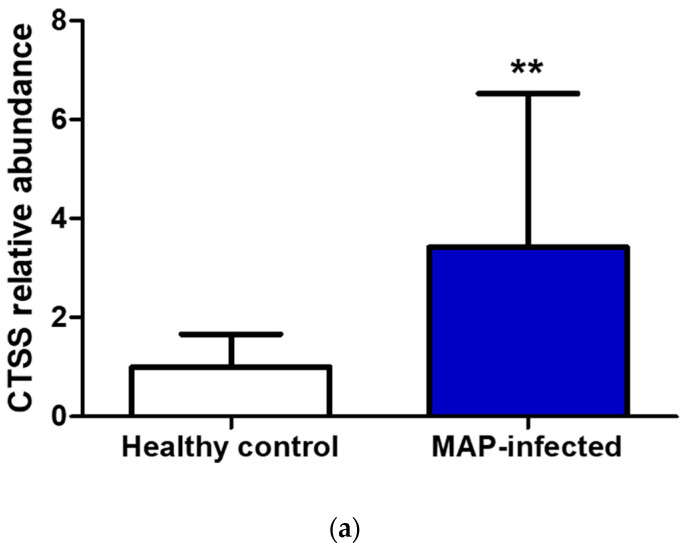

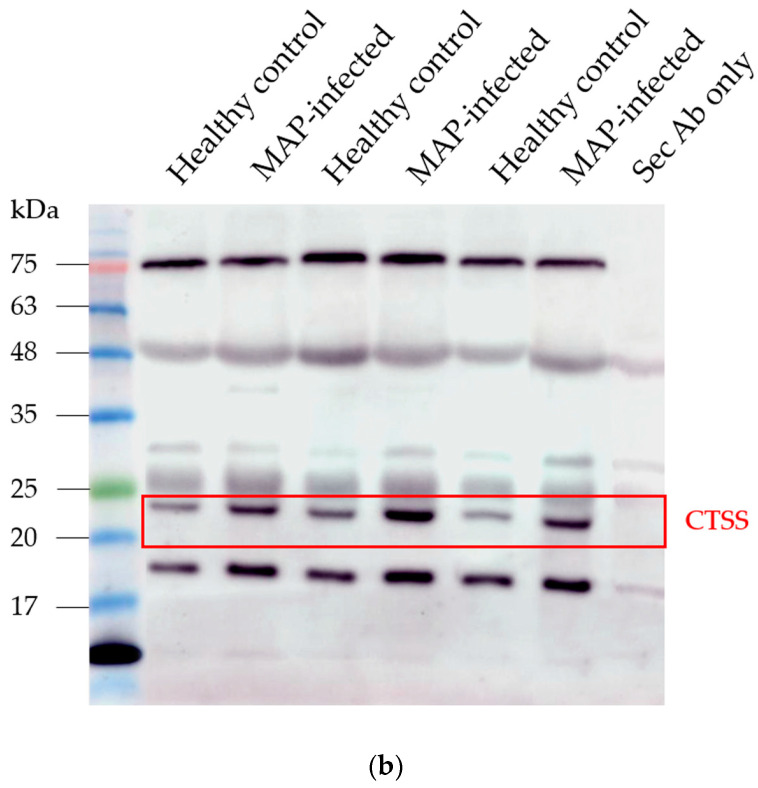
Elevated CTSS abundance was verified and quantified with Western blotting. (**a**) CTSS expression was significantly (** *p* ≤ 0.01) increased in serum of MAP-infected dairy cows (*n* = 12, blue column) compared to healthy controls (*n* = 9, white column, set to factor 1). (**b**) Representative Western blot showed higher abundance of CTSS in serum of MAP-infected dairy cows compared to healthy controls.

In the serum of MAP-infected dairy cows, we observed a 1.8 higher abundance of CTSS (*q* = 0.0001) compared to healthy controls from the same infected herd and a 1.5-fold increase in CTSS (*q* = 0.0005) compared to healthy controls from another uninfected herd using LC-MS/MS analysis. Confirmation of the observed elevated abundance of CTSS using Western blotting revealed a 3.4-fold enrichment of CTSS (*p* = 0.008) in the serum of MAP-infected dairy cows compared to healthy controls (Figure 5).

## 4. Discussion

Paratuberculosis is a severe and incurable disease that affects cattle and other ruminants, leading to significant economic losses [4,40,41]. Moreover, due to the spreading of MAP and its potential entry into the food chain, it poses a risk to human health [22]. Therefore, in accordance with the One Health approach, there is a pressing need to deepen our understanding of the immune response of cattle to MAP and to improve diagnostic methods for reliable disease detection. Serum is a commonly used biological material for diagnostic procedures and offers valuable insights into immune response through the examination of metabolites and proteins [42].

In this study, we identified differently abundant proteins in the serum of MAP-infected dairy cows and controls from herds with divergent MAP infection status. We assumed that the control group from the infected herd had been exposed to MAP but had remained uninfected, whereas the control group from the uninfected herd had never had contact with the pathogen. This allowed us to discern differences in host–pathogen interactions and detect immune reactions essential for the successful elimination of MAP.

The pathway enrichment analysis revealed an association of significantly higher abundant proteins with immune system-related pathways. Notably, the pathway “Fcgamma receptor (FCGR) dependent phagocytosis” and all its subordinated pathways “Regulation of actin dynamics for phagocytic cup formation”, “FCGR activation” and “Role of phospholipids in phagocytosis” were enriched in MAP-infected cows compared to healthy controls from the infected herd (Figure 3a). None of these pathways were enriched in comparison to the control group from the uninfected herd (Figure 3b). These findings suggest a potential influence of the “Fcgamma receptor (FCGR) dependent phagocytosis” pathway on susceptibility to bovine paratuberculosis.

Since MAP enters the organism through phagocytosis by macrophages, the inhibition of this pathway might hinder MAP persistence within the organism. This may be a crucial insight because the exact mechanisms enabling MAP survival within host cells remain elusive. The agent is suspected of persisting inside the phagosomes within these cells, as has already been proven for *Mycobacterium tuberculosis* (Mtb) [43,44]. The exact mechanisms enabling MAP survival are unknown, but it is assumed that MAP prevents maturation and acidification of the phagosome and blocks phagolysosome fusion, as shown for Mtb [43,44,45]. Consequently, the phagocytosis of MAP is a critical factor of infection.

The functional enrichment of “FCGR dependent phagocytosis” in MAP-infected cows compared to healthy cows from the infected herd showed that inhibition of this pathway might contribute to overcoming the persistence of MAP in the organism. In this enriched pathway group, the higher abundant protein FCGR1A, also known as CD64, in MAP-infected cows compared to both control groups (Figure 1), was described in several studies of *Mycobacteriaceae*-induced diseases [46,47,48,49]. In cattle, FCGR1A is expressed in leukocytes, especially in macrophages [50]. Soluble FCGR1A was detected in human serum [51]. We are the first to describe FCGR1A in bovine serum.

The function of FCGR1A in cattle has not been fully researched. However, in humans, it is involved in various immune effects, such as phagocytosis, production of reactive oxygen species, cytokine expression and antigen presentation [52]. Studies have demonstrated an increase in FCGR1A expression in the transcriptome of ileocaecal lymph nodes in sheep diseased with paratuberculosis [46]. So far, the significance of the increase in FCGR1A in bovine paratuberculosis is unknown. A study with tissue samples of cows with bovine tuberculosis caused by *Mycobacterium bovis* (Mb) showed the involvement of FCGR1A in the immune response against Mb [47]. In the peripheral blood of human children with intrathoracic tuberculosis induced by Mtb, FCGR1A levels correlated with the extent of the disease [48]. An increase in high-affinity FCGR1 expression could be induced with IFNγ in humans [53]. Based on an increased level of IFNγ in paratuberculosis-diseased cattle [54], we suspected an IFNγ-induced expression of FCGR1A in cattle and the extracellular shedding of soluble CD64.

Due to an improved control of Mtb in FCGR1-knockout mice [49] and a decrease in FCGR1A concentrations after anti-tuberculosis treatment in humans [48], we hypothesized that soluble FCGR1A had a negative impact on the immune response to MAP and facilitated their intracellular survival by interacting with soluble immune complexes and blocking Fc-dependent immune reactions [55]. Another soluble isoform of FCGR, soluble FCGR2b, inhibited antibody production in murine spleen cells [56], which could be transferred to soluble CD64, resulting in an additional impairment of adaptive immune response.

The effects of soluble FCGR1A in immune response, especially against MAP, should be explored more closely. Above all, new insights into its impact on susceptibility to bovine paratuberculosis could arise. However, in terms of its suitability as a specific biomarker for *Mycobacteriaceae*-induced diseases, especially bovine paratuberculosis, FCGR1A is not suitable due to its characterization as a diagnostic biomarker of infection and sepsis in humans [57,58].

Enriched pathway analysis provided an over-representation of “neutrophil degranulation” in MAP-infected cows compared to both healthy control groups (Figure 4a,b). The role of neutrophils in bovine paratuberculosis is still unknown. Neutrophils are highly effective cells of the innate immune system and kill microorganisms through various functions, including phagocytosis, degranulation, production of reactive oxygen species (ROS) and neutrophil extracellular trap (NET) release [59]. An increased migration of neutrophils in lesions at the early stages of the infection with MAP was shown in experimentally infected calves [60]. In a gene ontology analysis of long non-coding RNA target genes in macrophages of cattle with paratuberculosis, an enrichment of biological processes associated with neutrophils was found [61].

After analyzing the proteins involved in the enriched pathway “neutrophil degranulation” including peroxiredoxin 6 (PRDX6), cysteine-rich secretory protein 3 (CRISP3) and Cathepsin C (CTSC) (Table 1), it is evident that they cover various neutrophil functions. During neutrophil activation in humans, PRDX6 translocates to the plasma membrane and increases the production of ROS [62]. CRISP3 was found in granules of human neutrophils, which could suggest an antimicrobial role of CRISP3 in innate immune responses [63]. Furthermore, human CTSC is essential for optimal NET formation [64]. In a strong innate response to MAP, caprine neutrophils use all their effector functions—phagocytosis, chemotaxis, degranulation and NET release [65]. Oral vaccination against MAP in a rabbit infection model stimulated the phagocytosis rate of neutrophils and additionally increased NET release against MAP and non-related pathogens [66].

The involvement of neutrophils and their effector functions in early and advanced stages of bovine paratuberculosis is confirmed by these findings and the over-represented neutrophil degranulation in our pathway enrichment analysis. We put forward the hypothesis that the immune system of MAP-infected cattle attempts to combat the disease through increased activation of neutrophils, but the immunological functions of neutrophils are not effective in the host defense against MAP. MAP can escape the NETs by degrading NETs through an extracellular DNAse, which promotes the colonization of MAP and the formation of granulomas in mice [67]. Additionally, bovine neutrophils are effective in killing and defending against MAP, but in the presence of macrophages, the killing rate worsens, and levels of pro-inflammatory cytokines IL-1β and IL-8 are lower, leading to fewer pathological injuries [68].

These studies support our hypothesis and even describe a worsening of the disease due to neutrophil effector functions. Further research should analyze the mechanism of the host–pathogen immune response of neutrophils against MAP and their possible influence on the degradation of bovine paratuberculosis.

In our proteomics study, we detected a higher abundance of CTSS in the serum of MAP-infected dairy cows compared to both healthy control groups (Figure 1a,b). Pathway enrichment analysis revealed the involvement of CTSS in multiple enriched pathways. The involvement of CTSS in enriched pathways was observed in comparison to both healthy control groups (Table 1), leading us to select CTSS as a candidate for a biomarker in paratuberculosis-infected cattle. CTSS is a lysosomal cysteine peptidase, primarily found in immune cells, including professional antigen-presenting cells, B-cells, dendritic cells and macrophages. It plays a role in extracellular matrix remodeling and regulates MHC class II antigen presentation [69]. Thereby, CTSS proteolyzed the MHC class II-associated chaperone invariant chain Ii and produced class II invariant chain peptide (CLIP) [70,71]. The CLIP fragment is exchanged for antigenic peptide fragments, and the peptide-loaded MHC class II is transported to the plasma membrane where it will be recognized by CD4^+^ T cells [70,71].

In an in vitro study with human macrophages, infection with *Mycobacterium bovis* bacillus Calmette–Guérin (BCG) induced the inhibition of CTSS with IL-10 and consequently reduced MHC class II antigen presentation [72]. In contrast, the infection with a BCG strain that secreted active human CTSS led to an increased MHC class II mycobacterial antigen presentation on the surface of macrophages [73], which shows that BCG does not directly inhibit MHC class II presentation, but its expression is dependent on CTSS. In contrast to infection with lower pathogen vaccine strain BCG, we detected a higher abundance of CTSS in MAP-infected dairy cows (Figure 1a,b). We infer that the expression of CTSS could correlate with the pathogenicity of *Mycobacteriaceae*.

In addition to a higher abundance of CTSS, we could detect a higher abundance of bovine MHC class II (BOLA-DRA), which indicates a correlation of CTSS with expression of MHC class II antigen presentation. IL-10 expression by bovine macrophages was also reported in an in vitro infection assay with MAP, where neutralization of IL-10 resulted in an elevated killing rate of MAP, increased expression of tumor necrosis factor alpha, IL-8, IL-12 and MHC class II, higher rate of phagosome acidification and apoptotic cells [74]. This led to the hypothesis that IL-10 also inhibited the expression of CTSS in bovine macrophages, causing an inferior antigen presentation at the plasma membrane, phagosome acidification and apoptosis rate, where CTSS could be assigned to the corresponding pathways (Table 1). Interestingly, the pathway “MHC class II antigen presentation”, also associated with CTSS and BOLA-DRA, was enriched in the serum of MAP-infected cows compared to both healthy control groups (Table 1).

In vitro, MAP-infected bovine macrophages showed downregulation of MHC class II expression, which appears to be a strategy of MAP to secure their survival by inhibiting efficient mycobacterial antigen presentation to T cells [75]. In our previous in vitro study with PBMCs co-incubated with MAP, we observed a higher abundance of MHC class II complex proteins in MAP-resistant cows compared to persistently MAP-infected cattle [36], which describes an association of the expression of MHC class II with resistance to MAP. In our serum proteome analysis, we detected a higher abundance of BOLA-DRA in the serum of MAP-infected cows compared to both healthy control groups (Figure 1a,b).

The macrophages of mice infected with Mtb or BCG shedded MHC class II in plasma membrane-derived microvesicles and exosomes in an ATP-dependent manner [76]. Both organelles were able to present peptide-MHC class II complexes to T cells [76]. This study reveals an alternative process of an effective antigen presentation in mycobacterial infections. We hypothesize that in cattle infected with MAP, MHC class II is expressed at a lower level on the plasma membrane of antigen-presenting cells but is instead released in high amounts into the extracellular space for an alternative MAP-antigen presenting mechanism. Additional in vitro studies are necessary for characterizing the function of exosomes and microvesicles in mycobacterial antigen presentation in cows.

For the first time, we described a higher abundance of CTSS in serum in association with bovine paratuberculosis. We cannot yet explain why CTSS was secreted to a higher degree in the extracellular space in paratuberculosis-diseased cattle, but there could be a context of MHC class II in exosomes and microvesicles and the possible alternative mechanism of antigen presentation. So far, the mechanism of secretion and the function of extracellular CTSS in bovine host immune response to MAP are still unknown and need further investigation.

In our pathway enrichment analysis, CTSS, BOLA-DRA and FCGR1A revealed a strong association with immune system pathways, whereby we chose them as potential candidates. In relation to clinical practice, the comparison between MAP-infected dairy cows and healthy controls from the same infected herd was more relevant. In this group comparison, CTSS showed the highest *q*-value of all these three proteins. Therefore, we chose CTSS, which provides the best conditions for a potential biomarker for bovine paratuberculosis to be validated.

In our Western blot analysis, we loaded samples based on volume to validate our findings in a context relevant to potential practical applications. Although we performed normalization based on equal total protein content in our proteomics assay, we still observed a significantly higher abundance of CTSS in the serum of MAP-infected dairy cows using Western blotting (Figure 5a,b). We detected CTSS at a lower molecular weight, as expected, in recombinant CTSS expressed in *Escherichia coli* (*E. coli*). However, the molecular weight of naturally occurring proteins can be altered due to post-translational modifications [77], that do not occur in *E. coli*-expressed proteins [78]. The mature enzymatically active form of human CTSS has 24 kDa, lower than that of inactive CTSS [79]. Therefore, we hypothesized an enzymatic activity of CTSS secreted into the extracellular space.

Verification of CTSS using Western blotting promotes the practical utilization of extracellular CTSS as a potential biomarker for bovine paratuberculosis, including the development of a simple lateral flow assay. Nevertheless, there could be potential limitations in the use of CTSS as a biomarker regarding interindividual variability in MAP-infected dairy cows observed with Western blotting (Figure 5a) and the potential association of extracellular CTSS with other diseases. Therefore, it is essential to examine the expression of the protein in relation to the stage of the disease and its association with other mycobacterial- and non-mycobacterial-induced diseases in further investigations. So far, it is very rare that a biomarker cannot also be associated with other diseases [80].

Serum is an easily obtainable biological sample and reveals a high amount of information on the pathophysiological conditions in diseased animals [38]. However, the analysis of the serum proteome is challenged and limited due to the wide dynamic range of the proteins in it [38]. Compared to mass spectrometry, the proximity extension assay (PEA) as a novel technology has revealed a more sensitively targeted immunoassay [81]. However, due to the restricted offering of target products, PEA is not yet applicable for the analysis of bovine samples. Nevertheless, we will actively monitor advancements in technology and explore other emerging methodologies for their potential application in the analysis of bovine samples. As new technologies develop and become more accessible, we aim to reassess their suitability for the identification of better disease biomarkers.

Our quantified mass spectrometry analysis identified a total of 394 proteins in bovine serum, of which 70 were significantly differently abundant in the serum of MAP-infected dairy cows compared to healthy controls. A further study only revealed a significantly divergent abundance of eight proteins in the plasma of MAP-infected cattle [82]. In this study, differentially expressed proteins in a 2-dimensional fluorescence difference gel electrophoresis were analyzed with mass spectrometry [82]. This methodic procedure differed strongly from our enhanced label-free analyses and had, therefore, led to a limited number of identified proteins. Another serum proteomics study already detected 669 quantified proteins in bovine serum, of which only nine proteins showed significantly different abundances in MAP-infected cattle compared to the control group [83]. One of these nine proteins, fetuin B, also showed a significantly different abundance in our study. Due to the association of these already detected biomarkers with other diseases [84,85,86,87,88], there is still a need for suitable biomarkers of bovine paratuberculosis.

In clinical medicine, no single laboratory parameter should be used for the diagnosis of a disease without considering clinical parameters. However, a specific biomarker can contribute to an improvement in diagnostic tests. To ensure this, a cohort study with all already detected potential biomarkers for bovine paratuberculosis in serum or plasma needs to be conducted. It would also be interesting to research their correlation with other parameters, including disease stage and antibody levels. In addition to CTSS, our serum proteomics analysis revealed 69 other potential biomarkers that could also be further investigated, since it was shown that for some diseases they can be described through biomarker panels, not only one biomarker [89]. For the development of a suitable panel, our candidates identified in this study are available in PRIDE [90].

## 5. Conclusions

Our differential serum proteomics study reveals valuable new insights into the molecular mechanisms underlying host–pathogen interactions in MAP-infected cattle. Identifying CTSS as a potential biomarker provides a promising avenue for developing more effective diagnostic tools for bovine paratuberculosis, thereby contributing to efforts to combat bovine paratuberculosis.

## Figures and Tables

**Table 1:** Significantly (*q* ≤ 0.05) abundant proteins involved in significantly (*p* ≤ 0.05) enriched pathways in the serum of MAP-infected dairy cows compared to healthy controls from the same infected herd and also to another uninfected herd. Superordinate pathways consisting of one or more individual pathways were excluded.

Comparison Group	Enriched Pathway	Pathway Genes Total	Proteins
Healthy controls (Infected herd)	Pentose phosphate pathway	28	GPI, ALDOA
	Phagosome	160	ACTG1, FCGR1A, HLA-DRA, CTSS, TUBA4A, SFTPD
	Apoptosis	140	ACTG1, CTSC, SEPTIN4, CTSS, TUBA4A
	*Staphylococcus aureus* infection	98	FCGR1A, HLA-DRA, KRT25
	Tuberculosis	182	FCGR1A, HLA-DRA, LBP, CTSS
	Neutrophil degranulation	478	GPI, CRISP3, HP, ALDOA, PGLYRP1, PRDX6, CTSS, CTSC
	MHC class II antigen presentation	137	HLA-DRA, TUBA4A, CTSS, CTSC
	Regulation of actin dynamics for phagocytic cup formation	158	IGKC, FCGR1A, IGKV2-30, ACTG1
	Interferon gamma signaling	177	HLA-DRA, FCGR1A
	FCGR activation	103	IGKC, FCGR1A, IGKV2-30
	Role of phospholipids in phagocytosis	129	IGKC, FCGR1A, IGKV2-30
	Transfer of LPS from LBP carrier to CD14	3	LBP
	CD22 mediated BCR regulation	72	IGKC, IGKV2-30
	Gene and protein expression by JAK-STAT signaling after Interleukin-12	73	LCP1
	Butyrophilin (BTN) family interactions	12	PPL
	Classical antibody-mediated complement activation	97	IGKC, IGKV2-30
	Antigen activates B Cell Receptor (BCR) leading to generation of second messengers	103	IGKC, IGKV2-30
	Endosomal/Vacuolar pathway	15	CTSS
Uninfected herd	*Staphylococcus aureus* infection	98	KRT17, KRT14, FCGR1A, HLA-DRA, MASP1, KRT10, KRT25, KRT24
	Phagosome	160	ACTG1, TUBB, FCGR1A, HLA-DRA, CTSS, TUBB1, HLA-G, TUBA4A, SFTPD
	Estrogen signaling pathway	129	ADCY6, KRT17, KRT14, KRT10, HSPA1A, KRT25, KRT24
	Antigen processing and presentation	78	HLA-DRA, CTSS, HLA-G, HSPA1A
	Gap junction	87	ADCY6, TUBB, TUBB1, TUBA4A
	Viral myocarditis	69	ACTG1, HLA-DRA, HLA-G
	Gastric acid secretion	72	ADCY6, ACTG1, CA2
	Complement and coagulation cascades	82	PROCR, MASP1
	Apoptosis	140	ACTG1, CTSC, CTSS, TUBA4A
	*Salmonella* infection	250	TXN, ACTG1, TUBB, TUBB1, TUBA4A
	Neutrophil degranulation	478	SERPINA3, SERPINB1, TUBB5, CRISP3, COTL1, HBB, LYZ, PRDX6, CTSS, CTSC, HSPA1A
	MHC class II antigen presentation	137	TUBB5, TUBB1, HLA-DRA, TUBA4A, CTSS, CTSC
	Interferon gamma signaling	177	HLA-DRA, FCGR1A, HLA-G
	PKR-mediated signaling	88	TUBB5, TUBB1, TUBA4A, HSPA1A
	Endosomal/Vacuolar pathway	15	HLA-G, CTSS
	Regulation of Complement cascade	139	CFH, MASP1
	Gene and protein expression by JAK-STAT signaling after Interleukin-12 stimulation	73	LCP1

Column 1 (control group) indicates the control group to which the comparison was made. Column 2 (enriched pathway) contains the names of enriched pathways in ShinyGO analysis or in Reactome analysis. Column 3 (pathway genes total) displays the total number of genes in each pathway. Column 4 (proteins) shows the proteins in human orthologues gene names clustering to the enriched pathways.

## Data Availability

The mass spectrometry proteomics data presented in this study have been deposited to the ProteomeXchange Consortium via the PRIDE [90] partner repository (https://www.ebi.ac.uk/pride, accessed on 21 December 2023) with the dataset identifier PXD048042.

## Supplementary Materials

The following supporting information can be downloaded at https://www.mdpi.com/article/10.3390/metabo14040215/s1, Table S1: Schematic of the experimental design.
